# Parafoveal retinal function of epiretinal membrane foveoschisis: a pilot comparison among ERM phenotypes using MP-3 microperimetry

**DOI:** 10.1186/s12886-026-04726-8

**Published:** 2026-03-11

**Authors:** Reio Sekine, Tatsuya Jujo, Kota Kakehashi, Kaito Tomokiyo, Naoto Uchiyama, Naoto Tokuda, Hiroshi Toshida, Yasushi Kitaoka

**Affiliations:** Department of Ophthalmology, St. Marianna University School of Medicine, 2-16-1 Sugao, Miyamae-ku, Kawasaki, Kanagawa 216-8511 Japan

**Keywords:** Epiretinal membrane, Ectopic inner foveal layer, Epiretinal membrane foveoschisis, MP-3, Retinal sensitivity

## Abstract

**Purpose:**

To compare structural and functional outcomes among three epiretinal membrane (ERM) phenotypes—ERM without ectopic inner foveal layers (EIFL), ERM with EIFL, and ERM foveoschisis (ERM-F)—and to explore the anatomical and functional characteristics of ERM-F in a single-center, retrospective pilot study.

**Methods:**

Thirty-two eyes of 32 patients undergoing pars plana vitrectomy were categorized as ERM (*n* = 16), EIFL (*n* = 11), or ERM-F (*n* = 5). Swept-source OCT quantified central macular thickness (CMT) and morphology (cystoid spaces, retinal surface wrinkling, ellipsoid zone disruption, posterior vitreous detachment). Functional assessments included best-corrected visual acuity (BCVA, logMAR), parafoveal retinal sensitivity measured by MP-3 microperimetry within the central 5°, and M-CHARTS (horizontal/vertical). Assessments were performed at baseline and 1, 3, and 6 months postoperatively.

**Results:**

Baseline demographic characteristics, fixation stability, and metamorphopsia scores were similar across groups. Baseline BCVA and CMT were worse in EIFL than in ERM or ERM-F (both *p* < 0.05). Cystoid spaces were more frequent in EIFL and ERM-F than in ERM (*p* < 0.001). CMT decreased in ERM and EIFL (both *p* < 0.01) but not in ERM-F. BCVA improved postoperatively in the ERM and EIFL groups, and no significant intergroup differences in BCVA were observed at any postoperative time point. In contrast, MP-3 microperimetry demonstrated persistently lower retinal sensitivity in the EIFL group compared with the ERM group at all postoperative time points (all *p* < 0.05), while ERM-F showed parafoveal sensitivity numerically close to ERM by 6 months. Metamorphopsia (M-CHARTS) did not differ among groups; only ERM showed significant postoperative improvement in horizontal scores.

**Conclusions:**

Despite characteristic parafoveal schisis, ERM-F demonstrated postoperative BCVA and parafoveal sensitivity comparable to typical ERM, while EIFL remained functionally worse. MP-3 can reveal focal deficits not captured by BCVA, underscoring the value of multimodal assessment. As an exploratory, hypothesis-generating pilot study, these findings suggest that ERM-F may represent a relatively milder tractional phenotype and warrant confirmation in larger, prospective studies.

## Introduction

Epiretinal membrane (ERM) is a common age-related macular disorder characterized by the formation of a fibrocellular membrane on the internal limiting membrane, often resulting in visual dysfunction such as metamorphopsia and reduced visual acuity [1]. Although pars plana vitrectomy with membrane peeling generally yields favorable anatomical and visual outcomes, substantial interindividual variability exists in both preoperative functional impairment and postoperative recovery, underscoring the need for more refined prognostic stratification.

Advances in optical coherence tomography (OCT) have refined ERM phenotyping. The ectopic inner foveal layers (EIFL) concept proposed by Govetto et al. represents a progressive phenotype with inner retinal layer continuity and thickening and has been linked to poorer function and limited recovery [2]. In contrast, ERM foveoschisis (ERM-F)—characterized by parafoveal schisis-like spaces—has been described as a distinct OCT entity, separable from typical ERM as well as lamellar macular hole (LMH) and macular pseudohole [3]. Recent comparative studies have reported that ERM-F shows distinct OCT characteristics compared with typical ERM and other LMH–associated entities, and that surgical outcomes are generally favorable in terms of anatomical restoration, while functional recovery may vary across phenotypes [4, 5]. These reports support viewing ERM-F as an ERM-related configuration that warrants functional characterization within the ERM spectrum alongside EIFL-based staging.

Although ERM-F has been discussed within the spectrum of LMH–associated entities, prior reports have also framed it as an ERM-related tractional configuration within the broader ERM spectrum. Building on this concept, we aimed to clarify the functional positioning of ERM-F by comparing it with mild ERM without EIFL and advanced ERM with EIFL. Because pseudohole-like or LMH–like foveal contours can coexist with ERM-F, such mixed configurations were not used as exclusion criteria; eyes were included when they fulfilled our OCT criteria for ERM-F. Nonetheless, the functional profile of ERM-F—particularly parafoveal retinal sensitivity and metamorphopsia—remains incompletely characterized, and systematic comparisons with established ERM phenotypes, including EIFL-associated and mild ERM, are limited.

Beyond phenotype-based staging, contemporary OCT research has highlighted intraretinal biomarkers (e.g., hyperreflective retinal spots) that may inform ERM severity and progression, as well as postoperative functional recovery. Such biomarkers complement staging frameworks and may help explain discordance between postoperative best-corrected visual acuity (BCVA) and patient-reported quality of vision. We therefore positioned parafoveal microperimetry as a sensitive functional endpoint to be interpreted alongside OCT-derived intraretinal changes [6]. 

While best-corrected visual acuity (BCVA) remains a standard clinical endpoint, it may fail to capture localized parafoveal dysfunction and patient-perceived visual distortion, which are particularly relevant in tractional macular disorders. MP-3 microperimetry enables sensitive assessment of parafoveal retinal sensitivity within approximately 5° from fixation, which corresponds to the typical location of schisis in ERM-F, whereas M-CHARTS provides a quantitative measure of metamorphopsia. Together, these modalities offer complementary functional insights beyond conventional acuity-based evaluation.

This single-center, retrospective pilot study compared pre- and postoperative BCVA, central macular thickness (CMT), parafoveal retinal sensitivity (MP-3), and metamorphopsia (M-CHARTS) across three phenotypes—ERM without EIFL, ERM with EIFL, and ERM-F—to delineate the anatomical and functional characteristics of ERM-F. Given the limited sample size, this study was designed to be hypothesis-generating and to inform the design of future prospective investigations.

## Methods

### Study design and participants

This retrospective pilot observational study was approved by the Institutional Review Board of St. Marianna University School of Medicine (Approval No. 6716) and adhered to the tenets of the Declaration of Helsinki. Informed consent was obtained from all patients. We included 32 eyes of 32 patients diagnosed with idiopathic ERM who underwent pars plana vitrectomy between September 2023 and August 2024. Patients were categorized into three groups based on OCT morphology: ERM group (EIFL Stage 1–2, *n* = 16), EIFL group (EIFL Stage 3–4, *n* = 11), and ERM-F group (*n* = 5). During the study period, SS-OCT and MP-3 microperimetry were routinely performed, and we retrospectively enrolled consecutive eligible cases according to predefined inclusion and exclusion criteria.

### Exclusion criteria

Eyes with high myopia (axial length ≥ 26 mm), secondary ERM (due to diabetic retinopathy, uveitis, or retinal vascular disease), severe cataract (grade ≥ 3), or insufficient OCT image quality (signal strength ≤ 4) were excluded.

### OCT imaging and morphological assessment

Swept-source optical coherence tomography (SS-OCT; PLEX^®^ Elite 9000, Carl Zeiss Meditec, Dublin, CA, USA) was used to assess morphological features, including central macular thickness (CMT), presence of cystoid spaces, retinal surface wrinkling, ellipsoid zone (EZ) disruption, posterior vitreous detachment (PVD), and EIFL staging according to Govetto et al. Measurements were performed by multiple independent graders at baseline and at 1, 3, and 6 months postoperatively. CMT was defined as the mean retinal thickness within the central 1-mm ETDRS subfield centered on the fovea. Cystoid spaces were defined as discrete, well-demarcated hyporeflective intraretinal cavities on B-scan OCT, predominantly located within the inner nuclear layer and/or outer plexiform layer. EZ disruption was assessed on fovea-centered B-scan OCT images across the parafoveal region and was defined as a discontinuity or absence of the EZ band within the central 1-mm fovea-centered area; disruption was recorded as present if observed on any B-scan within this predefined area. In ERM-F eyes, schisis and cystoid spaces were recorded as separate entities: schisis was confined to the ONL–OPL (Henle’s fiber layer) splitting component, whereas cystoid spaces were defined as discrete intraretinal cystic cavities in other layers. Retinal surface wrinkling was recorded as present when undulation of the ILM/inner retinal surface was observed on any fovea-centered B-scan within the parafoveal region.

### Diagnostic criteria

EIFL was classified according to the staging system proposed by Govetto et al. [2]

**Fig. 1:**
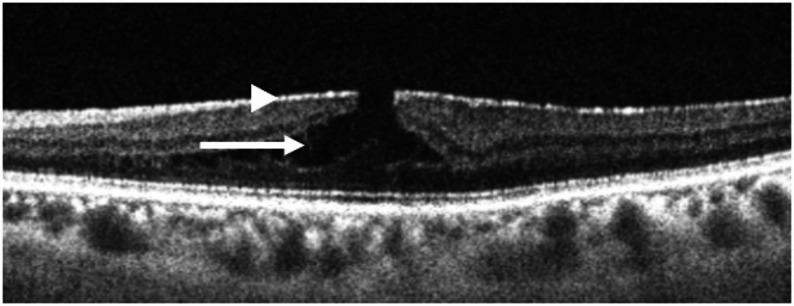
Diagnostic criteria of ERM foveoschisis. The diagnosis of ERM foveoschisis was made based on the presence of: (1) contractile ERM (arrowhead). (2) foveoschisis at the level of Henle’s fiber layer, typically between the ONL and OPL (arrow). ERM = epiretinal membrane; ONL = outer nuclear layer; OPL = outer plexiform layer

ERM-F was defined on swept-source OCT by the presence of a contractile epiretinal membrane accompanied by schisis-like splitting at Henle’s fiber layer (between the ONL and OPL), typically in the parafoveal region (Fig. 1), confirmed on at least two consecutive fovea-centered B-scan slices, and in the absence of tractional retinal detachment or a full-thickness macular hole [3]. Eyes with macular pseudohole or LMH–like foveal contours were not excluded if they met the OCT criteria for ERM-F.

Within the ERM-F group, 0/5 eyes showed mixed-type configurations meeting criteria for LMH-associated entities; however, 2/5 eyes had a macular pseudohole-like contour.

### Visual and functional assessments

**Fig. 2:**
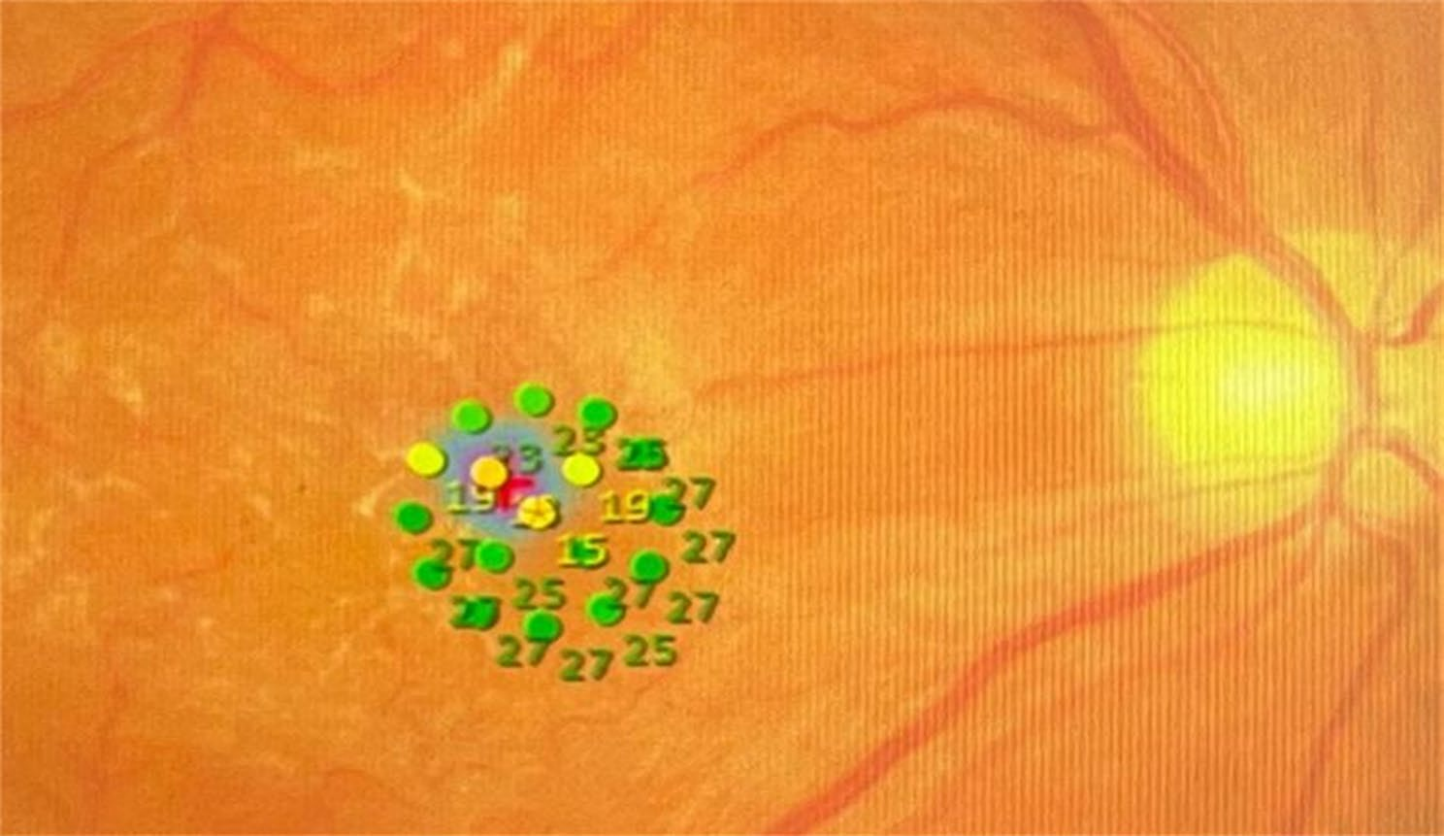
MP-3 microperimetry test pattern. Parafoveal retinal sensitivity was measured at 17 points within the central 5°. Numbers indicate retinal sensitivity values in decibels (dB)

BCVA was measured using a Landolt C chart and converted to logMAR units. Metamorphopsia was evaluated using horizontal and vertical M-CHARTS (Inami, Tokyo, Japan). Microperimetry was performed using MP-3 (NIDEK) with a 5°-17 point pattern (5 deg–17P-F) on a 31.4-asb white background, using a red single-cross fixation target (max 1.0°). Stimuli were Goldmann size III (white, 200 ms) with a 34-dB dynamic range using the 4–2 fast strategy; the Color Fundus, Refinement, Recheck, and Pre-test options were enabled (Fig. 2). Fixation stability (including the percentage of fixation points within 2° and 4° and BCEA) and false-positive/false-negative responses were recorded for each examination; all included tests showed stable fixation with minimal false-positive/false-negative responses.

### Surgical procedure

All surgeries were performed by two experienced vitreoretinal surgeons using 27-gauge microincision vitrectomy. ERM peeling was performed first, followed by internal limiting membrane (ILM) peeling in all cases. Combined cataract surgery was conducted in phakic eyes. No intraocular tamponade was used in any case. The extent of ILM peeling was not standardized in this retrospective study and was determined at the surgeon’s discretion. The ILM was stained with brilliant blue G in all cases to facilitate peeling.

### Statistical analysis

Statistical analyses were performed using JMP Pro (SAS Institute, Cary, NC, USA). Categorical variables were compared using Fisher’s exact test (Freeman–Halton extension for r×c tables), as appropriate. Continuous variables were compared using one-way ANOVA followed by Tukey–Kramer honestly significant difference (HSD) tests for post hoc pairwise comparisons. In addition, effect sizes are reported as between-group mean differences (dB) with 95% confidence intervals calculated using Welch’s method for key MP-3 comparisons (preoperative and 6 months).

The primary endpoint was the mean retinal sensitivity at 6 months. Between-group comparisons were interpreted primarily based on estimated differences with 95% confidence intervals; analyses at other timepoints and within-group changes were considered exploratory.

## Results

### Baseline characteristics

A total of 32 eyes from 32 patients were included in this study, comprising 16 eyes in the ERM group, 11 eyes in the EIFL group, and 5 eyes in the ERM-F group (Table 1).

**Table 1:** Baseline characteristics of patients in ERM, EIFL, and ERM-F groups

Characteristic	ERM (*n* = 16)	EIFL (*n* = 11)	ERM-F (*n* = 5)	*p*-value
Age, years (mean ± SD)	62.1 ± 9.6	67.5 ± 9.2	64.0 ± 10.4	0.36
Sex, Male (n, %)	7 (44%)	5 (45%)	2 (40%)	0.97
Sex, Female (n, %)	9 (56%)	6 (55%)	3 (60%)	
Axial length, mm (mean ± SD)	25.1 ± 1.4	24.8 ± 1.3	24.9 ± 1.2	0.90
Combined cataract surgery, n (%)	13 (81%)	11 (100%)	5 (100%)	0.25
Preoperative CMT, µm (mean ± SD)	411.6 ± 62.9	507.0 ± 110.2	375.4 ± 119.1	0.01*
Preoperative BCVA (logMAR, mean ± SD)	0.07 ± 0.11	0.41 ± 0.32	0.03 ± 0.06	0.0005*
Horizontal M-CHARTS (mean ± SD)	0.50 ± 0.60	0.71 ± 0.57	0.54 ± 0.50	0.64
Vertical M-CHARTS (mean ± SD)	0.33 ± 0.35	0.51 ± 0.44	0.44 ± 0.25	0.48
Fixation point F2 (%)	91.8 ± 10.5	90.3 ± 12.9	92.4 ± 8.0	0.91
Fixation point F4 (%)	98.4 ± 2.7	98.3 ± 3.2	99.6 ± 0.9	0.63

Continuous variables are presented as mean ± standard deviation (SD) and were compared using one-way analysis of variance (ANOVA). Categorical variables are presented as number (percentage) and were compared using Fisher’s exact test (Freeman–Halton extension for r×c tables). A p-value < 0.05 was considered statistically significant

The mean age was 62.1 ± 9.6 years in the ERM group, 67.5 ± 9.2 years in the EIFL group, and 64.0 ± 10.4 years in the ERM-F group (*p* = 0.36, ANOVA). Sex distribution was comparable (male: 44% in ERM, 45% in EIFL, 40% in ERM-F; *p* = 0.97, Fisher’s exact test), as was axial length (ERM: 25.1 ± 1.4 mm; EIFL: 24.8 ± 1.3 mm; ERM-F: 24.9 ± 1.2 mm; *p* = 0.90, ANOVA). Combined cataract surgery was performed in 13/16 eyes (81%) in the ERM group, 11/11 eyes (100%) in the EIFL group, and 5/5 eyes (100%) in the ERM-F group.

Preoperative CMT was significantly greater in the EIFL group (507.0 ± 110.2 μm) compared to the ERM (411.6 ± 62.9 μm; *p* = 0.04, HSD) and ERM-F groups (375.4 ± 119.1 μm; *p* = 0.03, HSD), while no significant difference was found between the ERM and ERM-F groups (*p* = 0.82, HSD).

Preoperative BCVA (logMAR) was significantly worse in the EIFL group (0.41 ± 0.32) compared to the ERM (0.07 ± 0.11; *p* < 0.001, HSD) and ERM-F groups (0.03 ± 0.06; *p* < 0.001, HSD), with no significant difference between the ERM and ERM-F groups (*p* = 0.76, HSD).

There were no significant intergroup differences in fixation stability (F2: ERM 91.8 ± 10.5%, EIFL 90.3 ± 12.9%, ERM-F 92.4 ± 8.0; F4: ERM 98.4 ± 2.7%, EIFL 98.3 ± 3.2%, ERM-F 99.6 ± 0.9) or metamorphopsia scores measured by horizontal and vertical M-CHARTS (horizontal: ERM 0.50 ± 0.60, EIFL 0.71 ± 0.57, ERM-F 0.54 ± 0.50; vertical: ERM 0.33 ± 0.35, EIFL 0.51 ± 0.44, ERM-F 0.44 ± 0.25; all *p* > 0.05, ANOVA).

### Baseline OCT morphology

The baseline OCT morphological characteristics are summarized in Table 2. PVD was observed in nearly all eyes, with no significant intergroup differences (ERM: 100%, EIFL: 91%, ERM-F: 100%; *p* = 0.50, Fisher’s exact test**)**.

**Fig. 3:**
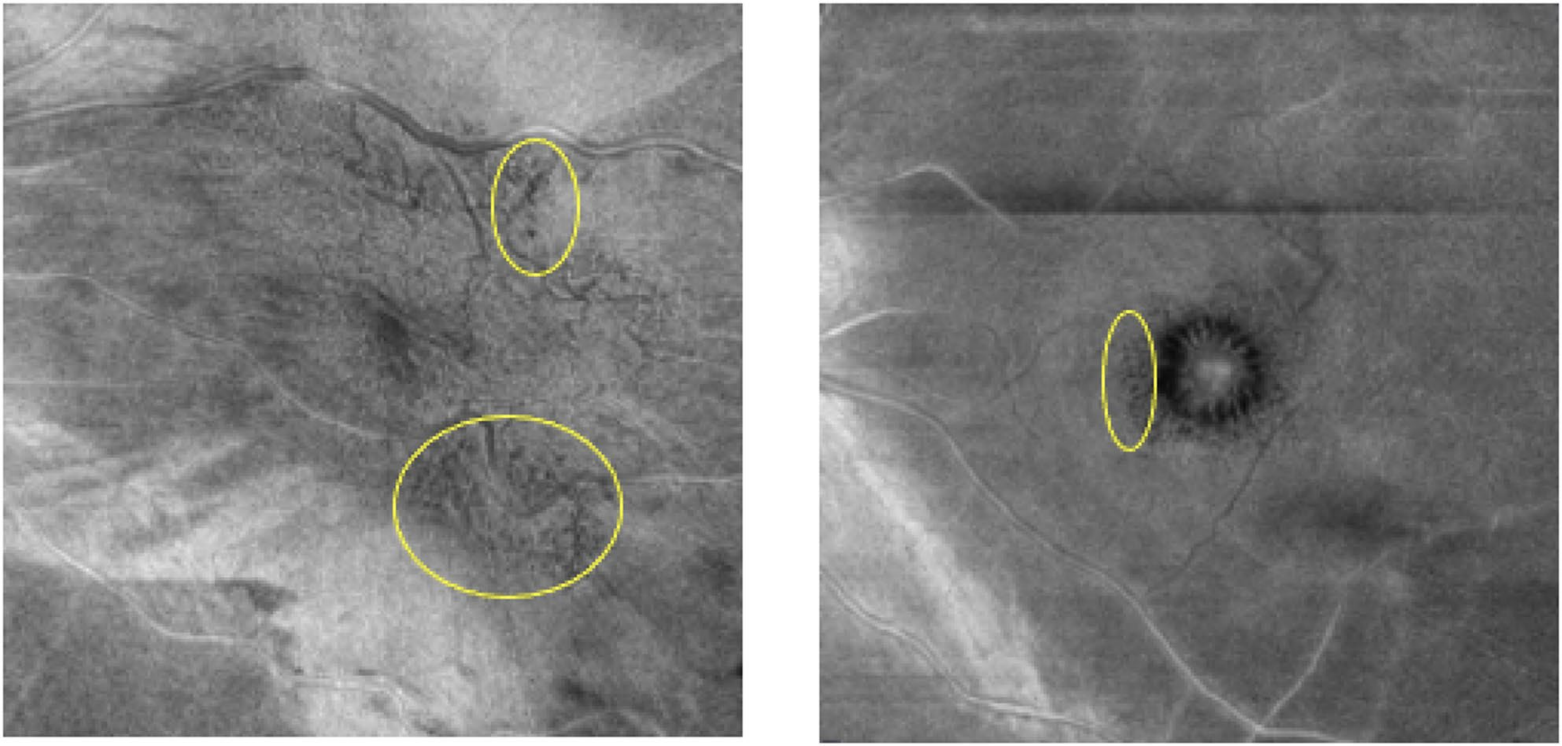
Distinct distribution patterns of cystoid spaces in EIFL and ERM-F. En face OCT images showing cystoid spaces (black dots) in eyes with ERM with EIFL (left panel) and ERM-F (right panel). In EIFL, cystoid spaces are irregularly distributed and tend to localize in areas of pronounced retinal contraction, independent of the foveal center. In contrast, ERM-F demonstrates a characteristic parafoveal arrangement, with cystoid spaces regularly distributed around the fovea

Cystoid spaces were significantly more frequent in the EIFL group (55%) and the ERM-F group (80%) compared to the ERM group (6%) (*p* < 0.001, Fisher’s exact test). Post hoc pairwise comparisons suggested differences between EIFL vs. ERM and ERM-F vs. ERM, whereas EIFL vs. ERM-F did not differ. In addition, representative en face OCT images illustrating differences in the distribution of cystoid spaces between ERM-F and EIFL are shown in Fig. 3. EIFL staging was applied to ERM and EIFL groups according to Govetto et al.; ERM-F eyes did not show EIFL and were therefore not staged.

**Table 2:** Baseline OCT morphological characteristics in ERM, EIFL, and ERM-F groups

OCT Finding	ERM (*n* = 16)	EIFL (*n* = 11)	ERM-F (*n* = 5)	*p*-value
PVD (+, %)	16 (100%)	10 (91%)	5 (100%)	0.50
Cystoid spaces (+, %)	1 (6%)	6 (55%)	4 (80%)	0.0008*
Retinal wrinkling (+, %)	3 (19%)	5 (45%)	4 (80%)	0.03*
EZ disruption (+, %)	1 (6%)	4 (36%)	0 (0%)	0.08
EIFL stage distribution	Stage 1: 37%	Stage 3: 72%	-	N/A
	Stage 2: 62%	Stage 4: 27%		

Categorical variables are presented as number (percentage) and were compared using Fisher’s exact test (Freeman–Halton extension for r×c tables), as appropriate. A p-value < 0.05 was considered statistically significant

Retinal surface wrinkling was observed in 19% of ERM eyes, 45% of EIFL eyes, and 80% of ERM-F eyes, showing a significant overall difference among groups (*p* = 0.03, Fisher’s exact test).

EZ disruption was present in 36% of EIFL eyes and 6% of ERM eyes, while none was observed in the ERM-F group. However, these differences did not reach statistical significance (*p* = 0.08, Fisher’s exact test).

Regarding EIFL stage distribution, the ERM group predominantly included Stage 1 (37%) and Stage 2 (63%) cases, the EIFL group consisted of Stage 3 (73%) and Stage 4 (27%) cases.

CMT significantly decreased over the 6-month follow-up in the ERM group (from 411.6 ± 62.9 μm to 300 ± 45 μm; *p* < 0.01, Wilcoxon signed-rank test) and the EIFL group (from 507.0 ± 110.2 μm to 330 ± 55 μm; *p* < 0.01, Wilcoxon signed-rank test), while no significant change was observed in the ERM-F group (from 375.4 ± 119.1 μm to 400 ± 50 μm; *p* = 0.12, Wilcoxon signed-rank test).

At 6 months postoperatively, the mean CMT did not significantly differ among the three groups (ERM: 300 ± 45 μm, EIFL: 330 ± 55 μm, ERM-F: 400 ± 50 μm; *p* = 0.07, ANOVA).

In the ERM-F group (*n* = 5), Henle’s fiber layer schisis at 6 months was classified as resolved in 3 eyes (60%) and improved in 2 eyes (40%), with no eyes showing persistence or worsening.

### BCVA and retinal sensitivity

**Fig. 4:**
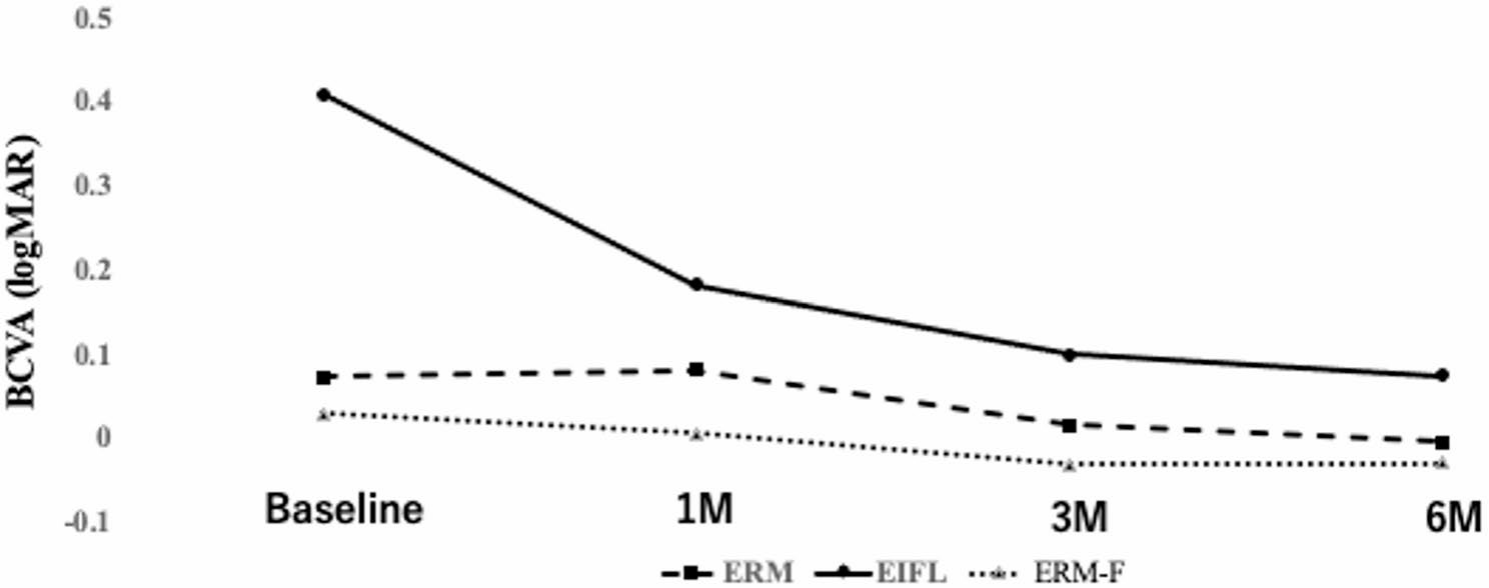
Postoperative changes in BCVA. Mean BCVA (logMAR) in the ERM, EIFL, and ERM-F groups over the 6-month postoperative follow-up period. At baseline, BCVA was worse in the EIFL group than in the ERM and ERM-F groups. Postoperatively, BCVA improved in the ERM and EIFL groups, and no apparent intergroup differences were observed at 1, 3, or 6 months. For clarity, error bars are omitted due to the small sample size. Time points indicate preoperative baseline and postoperative months 1, 3, and 6

The time course of BCVA (logMAR) over the 6-month postoperative period is shown in Fig. 4. At baseline, the EIFL group had significantly worse BCVA (0.41 ± 0.32) compared to the ERM (0.07 ± 0.11; *p* < 0.001, HSD) and ERM-F groups (0.03 ± 0.06; *p* = 0.006, HSD), with no significant difference between the ERM and ERM-F groups (*p* = 0.91, HSD).

Postoperative BCVA significantly improved in both the ERM and EIFL groups at 1, 3, and 6 months compared to baseline (all *p* < 0.05, Wilcoxon signed-rank test). In contrast, no significant change was observed in the ERM-F group over time. No significant intergroup differences in BCVA were detected at any time point (all *p* > 0.05, ANOVA).

**Fig. 5:**
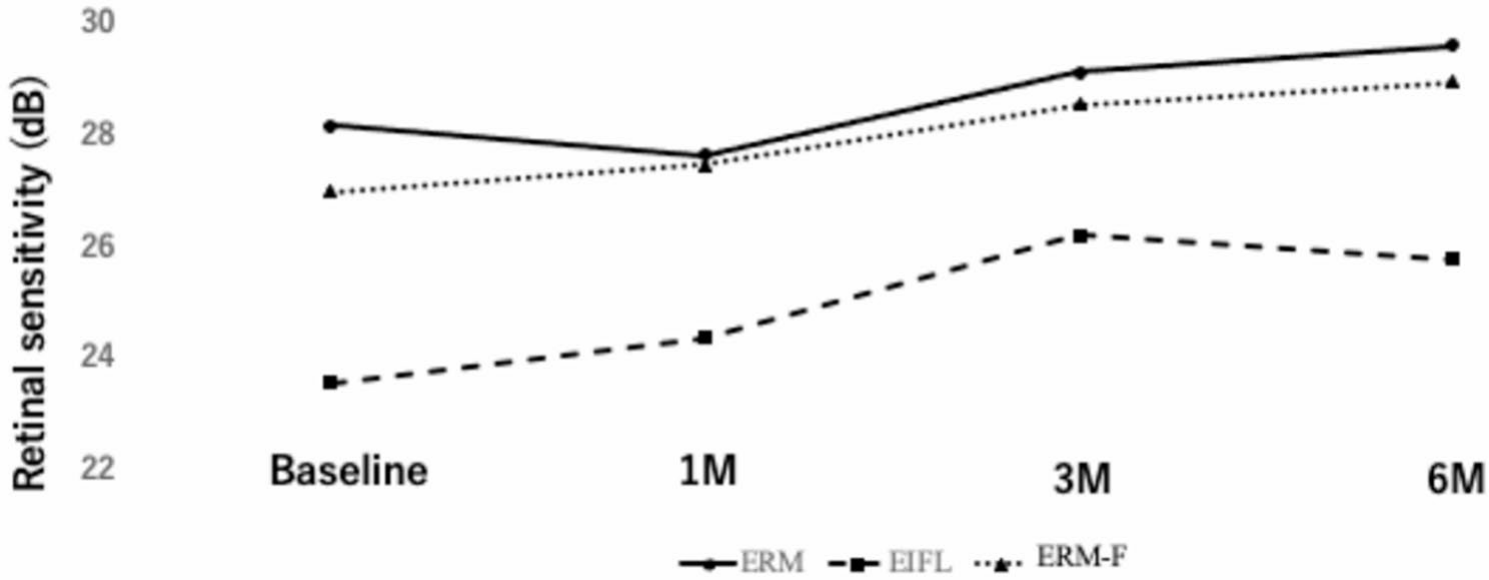
Time course of retinal sensitivity assessed by MP-3. Mean retinal sensitivity (dB) in the ERM, EIFL, and ERM-F groups over the 6-month postoperative follow-up. For clarity, error bars are omitted due to the small sample size. Post hoc pairwise comparisons (Tukey–Kramer HSD) indicated significant differences between the ERM and EIFL groups throughout follow-up (p < 0.05). Comparisons involving the ERM-F group did not reach statistical significance after Tukey–Kramer adjustment; however, effect sizes with 95% confidence intervals are provided in Table 4. Time points indicate preoperative baseline and postoperative months 1, 3, and 6

The mean retinal sensitivity over the 6-month postoperative period is shown in Fig. 5 and summarized in Tables 3 and 4. At baseline, the EIFL group had significantly lower retinal sensitivity (23.5 ± 4.5 dB) compared to the ERM group (28.1 ± 3.3 dB; *p* = 0.009, HSD), but not compared to the ERM-F group (27.0 ± 1.6 dB; *p* = 0.20, HSD), and no significant difference was found between the ERM and ERM-F groups (*p* = 0.80, HSD).

**Table 3:** Mean retinal sensitivity (dB) over time

Time Point	ERM (*n* = 16)	EIFL (*n* = 11)	ERM-F (*n* = 5)	*p*-value
Preoperative	28.1 ± 3.3	23.5 ± 4.5	27.0 ± 1.6	0.011*
1 Month	27.6 ± 1.7	24.4 ± 3.8	27.4 ± 2.2	0.012*
3 Months	29.1 ± 1.8	26.2 ± 2.1	28.5 ± 0.7	0.0016*
6 Months	29.6 ± 1.8	25.7 ± 3.5	28.9 ± 1.5	0.0021*

Data are presented as mean ± standard deviation (SD). Comparisons among groups were performed using one-way ANOVA. A p-value < 0.05 was considered statistically significant. Effect sizes for key pairwise comparisons are provided in Table 4

**Table 4:** Between-group mean differences in mean retinal sensitivity (MP-3, dB) with 95% confidence intervals (Welch)

Comparison	Preoperative mean difference (95% CI)	6-month mean difference (95% CI)
ERM − EIFL	4.60 (1.36 to 7.85)	3.81 (1.42 to 6.20)
ERM − ERM-F	1.18 (− 1.02 to 3.38)	0.63 (− 1.13 to 2.39)
ERM-F − EIFL	3.42 (0.26 to 6.59)	3.18 (0.60 to 5.76)

Values are mean differences (dB) with 95% confidence intervals calculated using Welch’s method

By 6 months, mean retinal sensitivity increased to 29.6 ± 1.8 dB in the ERM group, 25.7 ± 3.5 dB in the EIFL group, and 28.9 ± 1.5 dB in the ERM-F group (*p* = 0.002, ANOVA). Post hoc Tukey–Kramer HSD tests confirmed a significant difference between the ERM and EIFL groups (*p* = 0.002), whereas differences involving the ERM-F group did not reach statistical significance after adjustment (ERM vs. ERM-F, *p* = 0.87; EIFL vs. ERM-F, *p* = 0.06). To complement p-values, effect sizes with 95% confidence intervals are provided in Table 4; notably, the ERM vs. ERM-F difference at 6 months was small with a confidence interval spanning zero (mean difference, 0.63 dB; 95% CI, − 1.13 to 2.39), and these findings should not be interpreted as evidence of equivalence given the small ERM-F sample size (*n* = 5).

In the ERM-F group, postoperative OCT showed improvement in foveoschisis at the final follow-up, and mean MP-3 retinal sensitivity increased from baseline in all cases.

### Representative structural–functional course

**Fig. 6:**
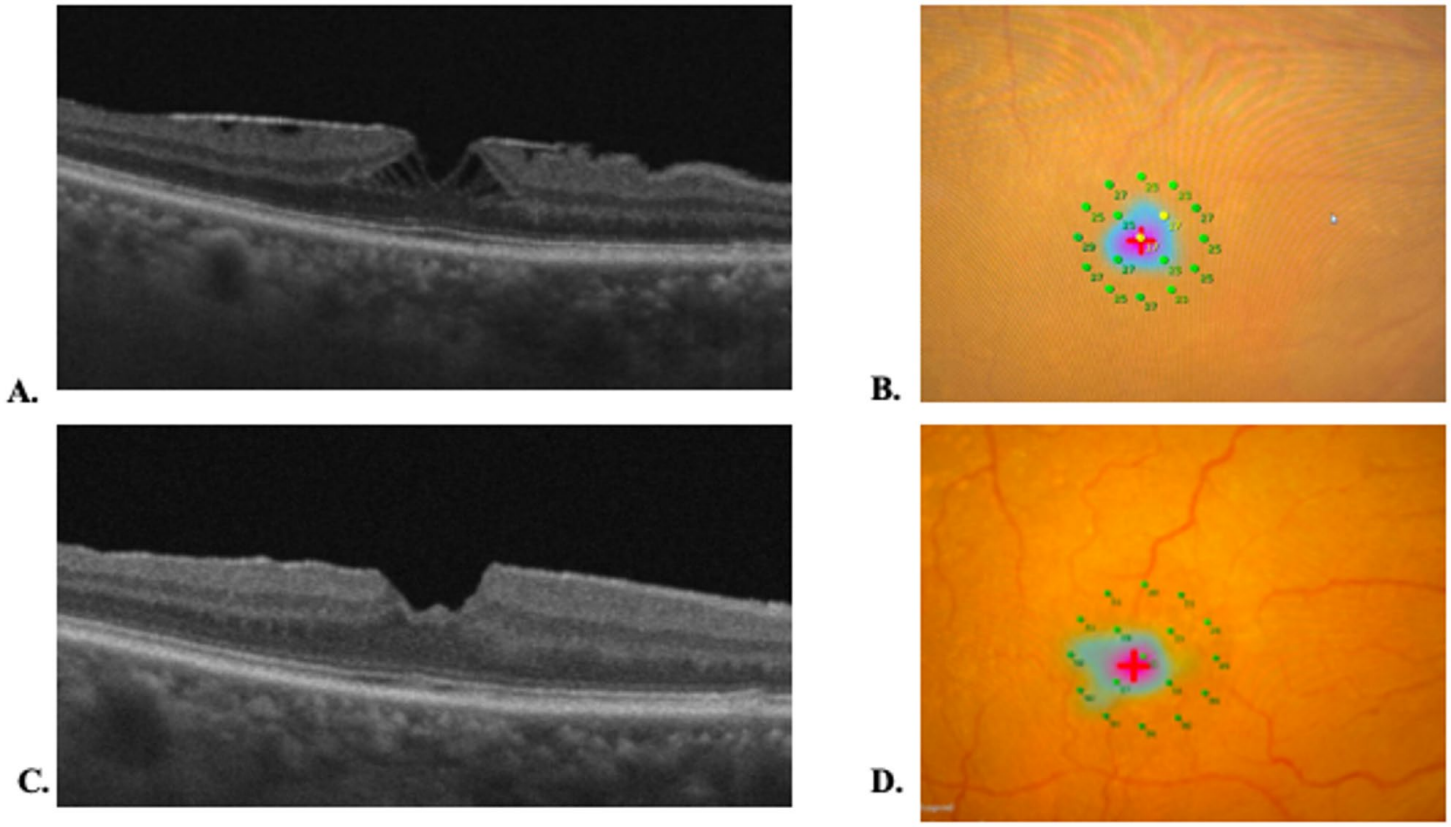
Representative ERM-foveoschisis case. **A**) Baseline OCT image of the right eye of a 76-year-old woman (axial length, 23.08 mm). Baseline logMAR BCVA was 0.00. ERM with foveoschisis and retinal wrinkling is observed; CMT was 422 μm. V-MCHARTS was 0.2. H-MCHARTS was 1.0. **B**) Baseline MP-3 microperimetry map is shown. **C**) At 6 months after surgery, logMAR BCVA improved to − 0.07. Foveoschisis is no longer evident on OCT, and CMT decreased to 386 μm. V-MCHARTS was 0. H-MCHARTS was 0. **D**)The corresponding MP-3 microperimetry map at 6 months is shown

**Fig. 7:**
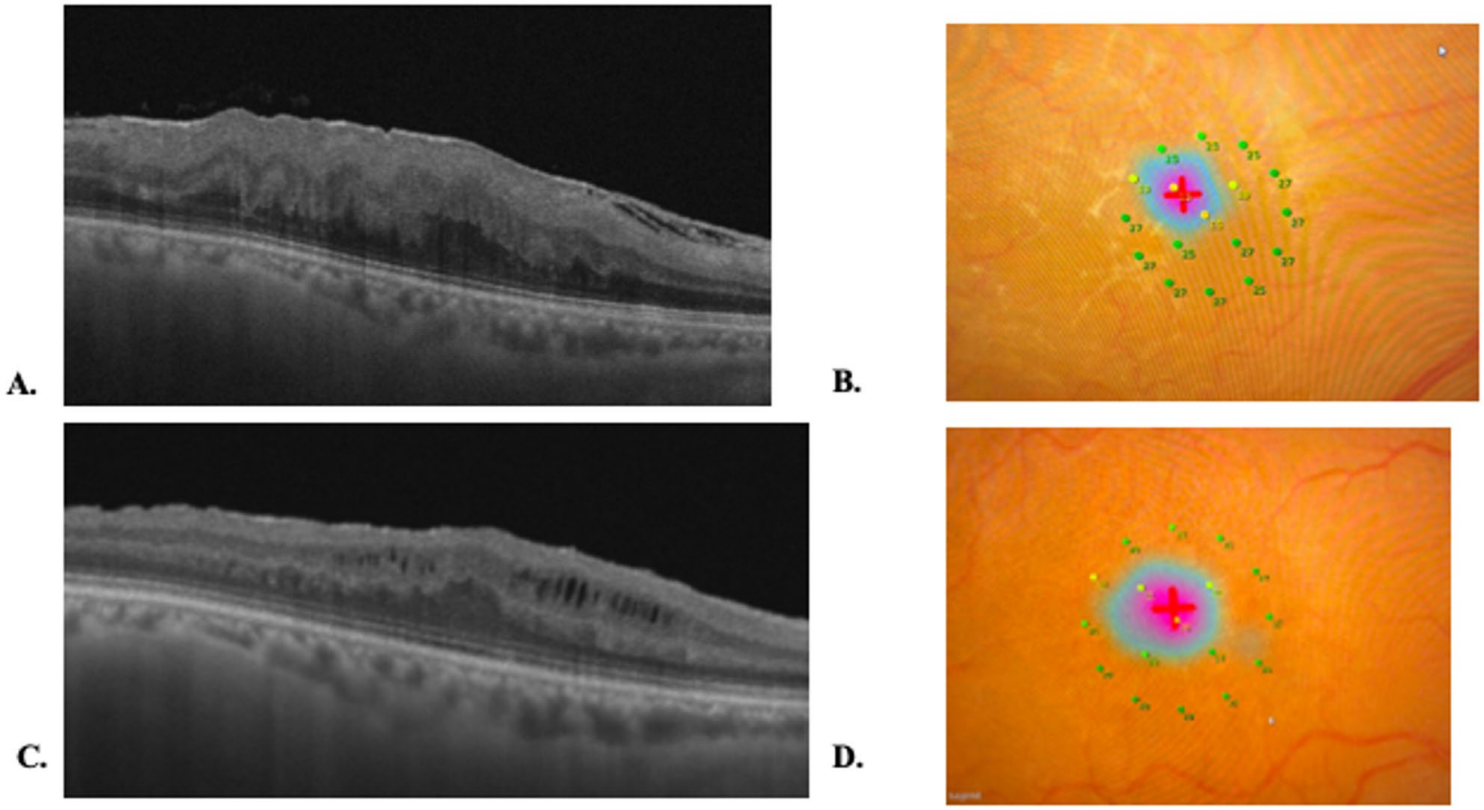
Representative ERM with EIFL case. **A**) Baseline OCT image of the right eye of a 66-year-old woman (axial length, 23.61 mm). Baseline logMAR BCVA was 0.22. ERM with EIFL is observed; CMT was 654 μm. V-MCHARTS was 1.1. H-MCHARTS was 0. **B**) Baseline MP-3 microperimetry map is shown. **C**) At 6 months after surgery, logMAR BCVA improved to 0.09. EIFL stage improved, but residual intraretinal cystoid spaces persisted at 6 months. CMT decreased to 435 μm. V-MCHARTS was 0.7. H-MCHARTS was 0. **D**) The corresponding MP-3 microperimetry map at 6 months is shown

Figure 6 shows a representative ERM-F case in which Henle’s fiber layer schisis resolved by 6 months after surgery, accompanied by improvement in parafoveal retinal sensitivity on MP-3. Figure 7 shows a representative EIFL case in which EIFL staging improved but residual intraretinal cystoid spaces persisted at 6 months

### Metamorphopsia

Metamorphopsia was assessed using horizontal and vertical M-CHARTS scores over the 6-month follow-up period. Only the ERM group showed a significant improvement in horizontal M-CHARTS scores postoperatively (from 0.50 ± 0.60 to 0.18 ± 0.31; *p* = 0.01, Wilcoxon signed-rank test). No significant improvements were observed in vertical M-CHARTS scores in the ERM group (from 0.33 ± 0.35 to 0.43 ± 0.51; *p* = 0.48) or in either horizontal or vertical scores in the EIFL group (horizontal: from 0.71 ± 0.57 to 0.50 ± 0.65; *p* = 0.31; vertical: from 0.51 ± 0.44 to 0.55 ± 0.43; *p* = 0.86) or the ERM-F group (horizontal: from 0.54 ± 0.50 to 0.22 ± 0.35; *p* = 0.46; vertical: from 0.44 ± 0.25 to 0.38 ± 0.24; *p* = 0.81).

Throughout follow-up, mean metamorphopsia scores tended to be higher in the EIFL group than in the ERM and ERM-F groups; however, no significant between-group differences were detected at any postoperative time point (all *p* > 0.05, ANOVA).

## Discussion

This single-center retrospective pilot study compared pre- and postoperative structural and functional parameters—BCVA, CMT, parafoveal retinal sensitivity measured by MP-3, and metamorphopsia on M-CHARTS—across three phenotypes: ERM without EIFL, ERM with EIFL, and ERM-F. Given its exploratory nature and limited sample size, the study was designed to be hypothesis-generating, with particular emphasis on delineating the anatomical and functional profile of ERM-F.

Modern OCT-based categorization of macular interface disease also considers epiretinal proliferation (EP/LHEP) and the broader tractional–degenerative continuum. Because ERM-F may overlap with, or be confused with, tractional LMH-spectrum phenotypes in real-world imaging, we applied layer-specific diagnostic criteria focusing on Henle’s fiber layer splitting and required the absence of a full-thickness macular hole; nevertheless, phenotypic overlap remains a consideration for external validity and should be addressed in larger validation cohorts [7]. 

In the surgical ERM spectrum, ERM-F has been reported to show favorable anatomical restoration after peeling, with functional recovery that may vary across cohorts and phenotypes. These prior observations are consistent with our pilot findings in which ERM-F showed postoperative microperimetry values closer to mild ERM than EIFL, while acknowledging inter-study heterogeneity and the limited sample size in our ERM-F subgroup.

### Baseline

No significant differences were observed in baseline characteristics, including age, sex, axial length, fixation stability, or metamorphopsia scores, among the three groups. However, the EIFL group exhibited significantly worse baseline BCVA and thicker CMT compared with both the ERM and ERM-F groups. Previous reports have shown no significant differences in baseline or postoperative visual acuity between typical ERM and ERM-F [4, 5]. However, no prior study has directly compared ERM-F with EIFL, which represents a more advanced stage of ERM pathology. In this study, the lack of significant difference in baseline BCVA between the ERM and ERM-F groups, and the significant difference between ERM-F and EIFL, suggests that ERM-F may represent a milder phenotype than EIFL-associated ERM.

Sasaki et al. reported that typical ERM cases tend to have significantly greater CMT than ERM-F [4]. Our study showed lower CMT in ERM-F than ERM with EIFL but not ERM without EIFL. Previous studies have also suggested a correlation between increased retinal thickness and worse visual prognosis in eyes with stage 3 ERM [8]. Although the small sample size in the ERM-F group, we could not do a correlation analysis between CMT and BCVA. Further study is needed.

### Retinal structure

In our structural OCT analysis, the frequency of cystoid spaces was significantly higher in both the EIFL and ERM-F groups compared to the ERM group. Previous studies have reported that widely distributed cystoid spaces are associated with a more advanced disease phenotype and worse visual prognosis [9]. In Fig. 3, although a quantitative spatial analysis of cystoid spaces was not performed, qualitative assessment of en face OCT images revealed distinct distribution patterns between ERM-F and EIFL. In the ERM-F group, cystoid spaces were frequently confined to the parafoveal region and showed a relatively regular, concentric arrangement around the fovea. In contrast, eyes with EIFL tended to exhibit more widespread and irregularly distributed cystoid spaces, often corresponding to areas of pronounced retinal contraction rather than being centered on the fovea.

In addition to mechanical distortion, persistently reduced microperimetric sensitivity in EIFL may also reflect reactive inner retinal remodeling. Recent work suggests that gliotic components within ERM and Müller cell–related changes correlate with EIFL thickness and postoperative recovery, supporting the hypothesis that advanced ERM/EIFL represents a transition from “predominantly tractional” changes toward reactive remodeling that may limit functional reversibility even after anatomically successful peeling [10]. 

These differences in cystoid spaces distribution may partly explain the observed functional discrepancies between groups and are consistent with previous reports indicating that broadly distributed cystoid changes are associated with more advanced disease phenotypes and poorer visual function.

Although there were no significant differences in visual function between the ERM-F and mild ERM groups, the presence of cystoid spaces differed. This discrepancy may reflect differences in tractional configuration and/or intraretinal remodeling across ERM phenotypes. It has been proposed that tangential traction is the force in typical ERM [11], whereas in ERM-F, the formation of cystoid spaces may be more influenced by anteroposterior traction, similar to that observed in myopic traction maculopathy (MTM). These mechanistic interpretations remain speculative because traction vectors were not directly quantified.

### Postoperative visual acuity and retinal sensitivity

Preoperatively, BCVA was significantly worse in the EIFL group than in the ERM and ERM-F groups; however, these between-group differences diminished after surgery, with no significant intergroup differences thereafter. By contrast, MP-3 microperimetry revealed persistent, significant differences in retinal sensitivity between EIFL and ERM at 1, 3, and 6 months, indicating that microperimetry captures functional deficits that BCVA alone may miss.

To our knowledge, this is the one of the first reports to quantify parafoveal retinal sensitivity in ERM-F. Prior work in myopic tractional maculopathy showed poorer microperimetric outcomes when schisis is present [12]. Although direct comparison is limited by concomitant myopic macular atrophy, it is plausible that foveoschisis in non-myopic eyes exhibits less functional loss than myopic foveoschisis or ERM with EIFL.

Microvascular findings support this gradient of functional impairment. In ERM, retinal vessel density correlates with postoperative retinal sensitivity [13], and eyes with EIFL show higher vessel density and a negative correlation with BCVA compared with eyes without EIFL [14]. Consistent with these reports, our data show persistently lower retinal sensitivity in EIFL relative to ERM, underscoring EIFL as a more advanced functional phenotype despite postoperative gains in BCVA.

### Parafoveal retinal sensitivity (MP-3)

Parafoveal retinal sensitivity measured by microperimetry provides complementary functional information beyond BCVA and may reflect residual dysfunction related to intraretinal microstructural integrity (e.g., inner retinal remodeling and microcystic changes).

Parafoveal sensitivity loss—often undetected by standard visual acuity testing—can affect patients’ subjective visual experience. In our cohort, ERM-F showed a slightly higher mean parafoveal retinal sensitivity than EIFL at 6 months postoperatively, suggesting that localized preservation or recovery of function may occur in ERM-F even when BCVA appears comparable.

To date, sensitivity profiling in ERM-F has been scarcely investigated. Our findings highlight retinal sensitivity as a complementary functional endpoint to BCVA. In particular, MP-3 microperimetry, which samples within 5° of the fovea, is well suited to visualize focal deficits attributable to schisis near Henle’s fiber layer and thereby captures subtle functional impairment that BCVA alone may miss.

Notably, in the ERM-F group, postoperative OCT showed improvement in foveoschisis accompanied by improvement in mean parafoveal retinal sensitivity on MP-3. While retinal sensitivity also tended to improve after surgery in the other phenotypes, this parallel structural and functional recovery in ERM-F is consistent with the concept that focal schisis-related dysfunction can be captured by microperimetry. Larger studies with sufficient variability in postoperative structural changes will be required to formally quantify structure–function relationships.

### Metamorphopsia

To our knowledge, this is the first study to compare metamorphopsia across three distinct phenotypes—ERM-F, mild ERM, and ERM with EIFL—using M-CHARTS. Prior work has shown that the maximum depth of retinal folds within the parafovea (MDRF) correlates with metamorphopsia in ERM-F and macular pseudohole [15], that MDRF is associated with α-SMA expression [16], and that the degree of MDRF may relate to postoperative visual outcomes [17]. These reports collectively implicate traction-related inner retinal deformation as a structural substrate for metamorphopsia.

In our cohort, M-CHARTS scores did not differ significantly among the three groups, and only the ERM group showed a significant postoperative improvement from baseline. Although MDRF was not measured and sample size was limited, retinal surface wrinkling tended to be more frequent in the ERM-F and EIFL groups, consistent with persistent tractional changes that may sustain metamorphopsia. Taken together with our findings on visual acuity and retinal sensitivity—where ERM-F did not show worse outcomes than EIFL—these results suggest that metamorphopsia in ERM-F may persist at similar levels to that in EIFL, despite relatively preserved anatomy and function.

Clinically, this implies that ERM-F should not be excluded from surgical consideration solely on the assumption of a “mild” phenotype; patient-reported distortion may persist even when BCVA and microperimetry are relatively favorable, and symptom-directed decision-making remains important.

The lack of significant between-group differences in M-CHARTS may reflect limited power and test variability, as well as persistent inner retinal distortion despite BCVA gains. Because fold metrics (e.g., MDRF) were not quantified, mechanistic interpretations should be considered hypothesis-generating.

## Conclusion

This single-center, retrospective pilot study compared clinical characteristics and functional outcomes across three ERM phenotypes—typical ERM, ERM with EIFL, and ERM-F. Although the EIFL group exhibited significantly worse preoperative BCVA and greater CMT, the ERM-F group—despite characteristic parafoveal schisis on OCT—achieved postoperative visual acuity and retinal sensitivity that was not evidently worse than typical ERM. MP-3 microperimetry provided functional information beyond BCVA and highlighted persistent parafoveal sensitivity deficits in EIFL, supporting the view that ERM-F may represents a relatively milder tractional phenotype than EIFL. Notably, this appears to be the first comparison of metamorphopsia across these three phenotypes, suggesting that ERM-F may show similar levels of metamorphopsia comparable to EIFL despite relatively preserved anatomy and microperimetry. Taken together, these exploratory, hypothesis-generating findings highlight the value of multimodal assessment—including microperimetry—when classifying ERM subtypes and considering surgical indications, and they motivate larger, prospective studies to validate and extend these results.

Clinically, in advanced ERM with EIFL, postoperative BCVA improvement may overestimate functional recovery, whereas microperimetry can reveal persistent parafoveal impairment that may be more relevant to symptoms and quality of vision. This supports the utility of MP-3 as a complementary functional endpoint in phenotype-based ERM assessment.

### Limitations

This single-center, retrospective pilot study has several limitations. The ERM-F cohort was small (*n* = 5), limiting statistical power and increasing the risk of type II error; thus, all findings should be interpreted cautiously. Follow-up was limited to 6 months. Potential confounders include lens status and combined cataract surgery, non-standardized extent of ILM peeling, and microperimetry measurement variability related to testing conditions and postoperative media clarity. We did not apply formal longitudinal mixed-effects modeling because the very small ERM-F sample could yield unstable estimates. Accordingly, these results are hypothesis-generating and should be validated in larger, multicenter prospective studies with standardized procedures and longer follow-up.

## Data Availability

The datasets generated and/or analyzed during the current study are not publicly available due to institutional ethics restrictions and the protection of patient privacy, but are available from the corresponding author on reasonable request.
